# Epidemiology of Antimicrobial Resistance Genes in *Streptococcus agalactiae* Sequences from a Public Database in a One Health Perspective

**DOI:** 10.3390/antibiotics11091236

**Published:** 2022-09-12

**Authors:** Gabriele Meroni, Valerio M. Sora, Piera Anna Martino, Alice Sbernini, Giulia Laterza, Francesca Zaghen, Alessio Soggiu, Alfonso Zecconi

**Affiliations:** 1 One Health Unit, Department of Biomedical, Surgical and Dental Sciences, School of Medicine, University of Milan, Via Pascal 36, 20133 Milan, Italy; 2 Department of Clinical and Community Sciences, School of Medicine, University of Milan, Via Celoria 22, 20133 Milan, Italy

**Keywords:** *S. agalactiae*, GBS infections, One Health, antimicrobial resistance, molecular epidemiology

## Abstract

*Streptococcus agalactiae* is a well-known pathogen in humans and food-producing animals. Therefore, this bacterium is a paradigmatic example of a pathogen to be controlled by a One Health approach. Indeed, the zoonotic and reverse-zoonotic potential of the bacteria, the prevalence of Group B Streptococci (GBS) diseases in both human and animal domains, and the threatening global situation on GBS antibiotic resistance make these bacteria an important target for control programs. An epidemiological analysis using a public database containing sequences of *S. agalactiae* from all over the world was conducted to evaluate the frequency and evolution of antibiotic resistance genes in those isolates. The database we considered (NCBI pathogen detection isolate browser—NPDIB) is maintained on a voluntary basis. Therefore, it does not follow strict epidemiological criteria. However, it may be considered representative of the bacterial population related to human diseases. The results showed that the number of reported sequences increased largely in the last four years, and about 50% are of European origin. The frequency data and the cluster analysis showed that the AMR genes increased in frequency in recent years and suggest the importance of verifying the application of prudent protocols for antimicrobials in areas with an increasing frequency of GBS infections both in human and veterinary medicine.

## 1. Introduction

*Streptococcus agalactiae*, also known as Group B *Streptococcus* (GBS), is a Gram-positive bacterium able to colonize healthy adults’ intestinal and vaginal tracts. Based on the capsular polysaccharide, *S. agalactiae* was categorized into ten serotypes (Ia, Ib, II, III, IV, V, VI, VII, VIII, IX). The disease-causing dominant serotype varies by geography and may have a different distribution of invasive and colonizing strains. The most prevalent serotypes associated with colonizing strains are serotypes I–V, whereas serotype Ia is the principal cause of maternal GBS disease. Additionally, a strong association between serotype and invasive infant disease has been observed regarding serotypes III, Ia, Ib, II, and V. Whereas, serotypes Ia, Ib, II, and V are the most prevalent causes of invasive disease in non-pregnant adults [1,2,3].

This commensal bacterium is the leading cause of invasive neonatal infections (e.g., bacteremia and meningitis) in industrialized countries and a pathogen of growing importance in the elderly, particularly in people having pre-existing diseases such as diabetes or cancer [1,2,3]. The most common syndromes due to invasive GBS disease in adults are bacteremia without a focus and skin/soft tissue infections, but bacteremia may also lead to seeding of the cardiac valves, and to endocarditis. Due to the burden of GBS disease in neonates, preventative measures have been developed to minimize invasive disease. In 1973, a program of maternal antibiotic administration began to prevent neonatal GBS disease [2]. Despite the effectiveness of Intrapartum Antibiotic Prophylaxis (IAP), according to the World Health Organization, GBS still causes 150,000 stillbirths and infant deaths worldwide [4]. Depending on the established guidelines, the administration of IAP is based on risk factors or screening protocols and, for this reason, antibiotic resistance rate monitoring is of paramount importance. The first-line antibiotic for IAP is penicillin. In cases of severe penicillin allergies, the recommendation is to use second-line antibiotics such as macrolides (erythromycin) and lincosamides (clindamycin); unfortunately, increasing resistance to both antibiotics restricts their use. While penicillin is still considered effective against GBS, increasing reports of isolates less sensitive to this antibiotic are causing great concern, especially when resistance to second-line antibiotics becomes an established trait in GBS [3,5]. Indeed, the spread of these resistant pathogens in humans and animals, and the potential contamination of the environment, through the use of manure as fertilizer will increase the global risk from a One Health perspective [6].

*S. agalactiae* is also a well-known pathogen in food-producing animals. Indeed, it is a leading cause of contagious mastitis in dairy cows [7,8,9], and streptococcosis leads to several clinical diseases in fish species in the aquaculture industry [10,11]. The *S. agalactiae* infection in cows has been considered a zoonotic disease in several countries. Moreover, the risk of transmission from humans to cows has also been suggested (reverse zoonosis) [12,13]. These aspects need further investigation, but they are not the only ones to be considered. Indeed, the control strategies of GBS in human and veterinary medicine are based mainly on antimicrobials and often with the same type of molecules. Therefore, the increasing incidence of antibiotic resistance poses a serious threat to disease treatment and is now recognized as a significant public health concern [14,15].

The *S. agalactiae* infections in human beings and animals are a paradigmatic example of a disease to be controlled by a One Health approach. Indeed, the zoonotic and reverse-zoonotic potential of the bacteria, the prevalence of GBS diseases in both human and animal domains, and the threatening global situation on GBS antibiotic resistance make these bacteria an important target for control programs. These programs should consider the bacteria’s genetic and phenotypic characteristics and the epidemiology of the infection with an approach that should consider both isolates from human beings and animals and the potential risk of spreading bacteria and resistance genes through the environment. 

Within a project aiming to develop effective control programs for *S. agalactiae*, we performed an epidemiological analysis using a public database (NCBI Pathogen Detection Isolate Browser; NPDIB) containing sequences of *S. agalactiae* from all over the world. This analysis aims to evaluate the frequency and how it changed in recent years, as well as the geographical and biological sources of antibiotic resistance genes in those isolates, to identify the potential presence of specific AMR patterns related to any of the considered factors. 

## 2. Results

### 2.1. Data Description

The public database at the date 30 April 2022 included 1828 isolates. Among them, 1164 were classified as clinical, 658 were not classified, and six were from environmental sources. From 2010 to 2015, when approximately 33% isolates were recorded, the overall number of isolates reported into the database increased at a consistent rate until 2020, when a rise in records of about 25% was noticed in less than two years (Table 1). The pattern was different for clinical isolates that showed a more erratic trend with the highest frequencies in 2011–2015 and 2019–2020 when nearly 60% of the clinical isolates were reported, while only 1% of the clinical isolates were reported in 2010–2012.

European isolates represent nearly 40% of all records, while African and Australian showed the lowest frequencies (<2%). The highest frequency of clinical isolates was also from Europe with 47.5% of the isolates, while the other geographical areas contributed about 10% each of the sequences, apart from Africa and Australia that showed very low frequencies in this case (Table 2).

Blood was the most frequent biological source, with 16% of the overall isolates and 25% of the clinical ones (Table 3). Interestingly, human milk was reported in 10% of the overall samples, and only in 2.9% of the clinical ones, while all other sources increased their frequency when clinical cases were considered. Animal sources represented less than 1% of the reported isolates. 

### 2.2. Resistance Gene Distribution

*S. agalactiae* is known to have multiple antimicrobial resistance (AMR) genes. Overall, 19 different AMR genes were considered in the database, and their frequency ranged from 0.5% of *catTC* and *ermT* up to 59.8% of *tetM* (Table 4). However, the clinical isolates showed a different distribution. Indeed, *catTC* and *ermT* were the genes with the lowest frequency (0.8%), while *tetM* also in this group had the highest frequency (66.5%). A detailed description of AMR associated with the genes is reported in Supplementary Table S1.

A statistical analysis (χ^2^ test) of the eight most frequently isolated genes (>100 positive sequences) was carried out to see whether there may be significant variations in the distribution pattern between overall sequences and clinical ones. The results showed that only *tetO* and *ant(6)la* significantly differed in frequency (Table 5). Indeed, *ant(6)la* had a higher-than-expected frequency in clinical cases, while *tetO* had a lower-than-expected frequency in the same group of sequences.

### 2.3. Cluster Analysis

**Figure 1:**
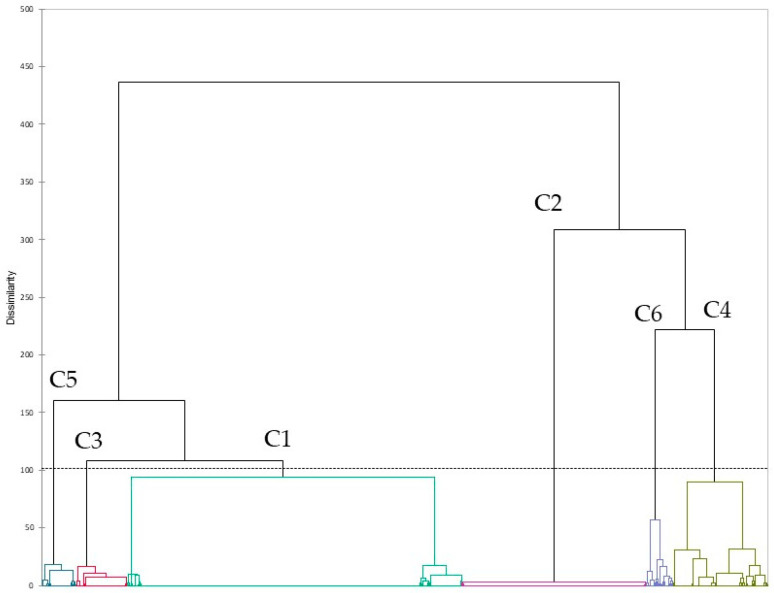
Dendrogram obtained from cluster analysis of the 1824 sequences based on antimicrobial genes frequency.

The AMR pattern of a microorganism is the result of the different combinations of AMR genes, and of their expression in the host. Cluster analysis (Figure 1), as described in Section 4, was performed to analyze the presence of these patterns and the relationship with the other available factors (year, area and source). 

The analysis identified six different clusters with a combination of the 19 different AMR genes. The numerosity of each cluster is the following: C1 (n = 843), C2 (n = 465), C3 (n = 128), C4 (n = 237), C5 (n = 86), and C6 (n = 69).

Table 6 reported the distribution of the 10 genes with a frequency >50 among the 6 clusters. Cluster 2 represents the isolates with the lowest AMR risk since none of the considered genes was reported. On the other hand, Cluster 6 includes all positive sequences for *lnuB* and *lsaE*, which were nearly absent in all other clusters. Among the other clusters, C1 includes sequences positive for *tetM*, *ermB* and *ant(6)la* with prevalences of 100%, 12% and 3 %, respectively, while the other seven genes were not found. Clusters 3, 4 and 5 showed a different distribution of AMR gene frequency. Cluster 3 may be characterized by a relative high frequency of *tetM* and *ermA*; cluster 4 by the presence of eight out of 10 the genes, and a high frequency for *tetO*; cluster 5 by the presence of six out of 10 genes, and a high frequency for *mrsD* and *tetM* (99% and 85%, respectively).

### 2.4. Association of Gene-Clusters and Isolate Characteristics

#### 2.4.1. Year of Reporting

Statistical analysis was performed to identify the association between cluster and the characteristics of the sequences (year, area and source of reporting). Table 7 shows that the frequency of cluster 2 (absence of AMR genes) was significantly less than expected after 2019, while cluster 6 (highest frequency of AMR genes) was absent until 2015. Then, its frequency started to increase with a peak in the period 2019–2020 (significant at Fisher’s exact test). Clusters 1 and 2 showed a declining trend, while cluster 5 significantly increased its frequency in 2019–2022.

The same analysis was applied only to isolates from clinical sources (Table 8), and some differences were observed compared to whole database analysis. Indeed, cluster 4 had a significantly higher-than-expected frequency in the period 2019–2022. The same result was observed for clusters 5 and 6, even if the differences were not significant for the period 2021–2022. Cluster 2 showed a significantly higher than expected frequency in the period 2021–2022.

#### 2.4.2. Area of Reporting

The analysis of the association between clusters and area of reporting (Table 9) showed that cluster 6 has a significantly higher-than-expected frequency in the Asian countries, while it is scarcely identified in Europe, despite the larger part of the sequence being European. In this area, a significantly high frequency was observed for cluster 4, while a lower-than-expected frequency was observed for cluster 2.

The analysis performed on clinical isolates (Table 10) gave similar results but few important differences. Indeed, cluster 1 was highly frequent in Europe, whereas cluster 2 had a lower-than-expected frequency in the same area, while its frequency was higher-than-expected in Americas.

#### 2.4.3. Source of the Isolates

The association between clusters and sequence sources (Table 11 and Table 12) showed that cluster 2 has a higher than expected proportion in milk samples, while it was lower than expected in blood. Isolates from the vagina, rectum, urine, and feces had a higher than expected frequency of sequences included in cluster 6. In the clinical subset, the higher frequency of cluster 6 was confirmed at least for isolates from urine and feces, while a higher-than-expected proportion of sequences included in cluster 3 was observed in blood isolates.

## 3. Discussion

### 3.1. Relevance of the Dataset

The database we considered (NCBI pathogen detection isolate browser) is a subset of a larger one, including 53 different bacteria and over a million sequences. Due to the voluntary nature of this information source, it does not strictly adhere to epidemiological standards (i.e., random sampling). However, the large number of sequences of *S. agalactiae* included in the database may be considered representative of the population of these bacteria related to human diseases, and it has practical importance including sequences from all over the world and from different biological sources. The very low frequency of sequences from animal sources should be considered a critical point from a One Health approach since the lack of these sequences cannot be related to low prevalence of infections in animals. Indeed, livestock-associated GBS infections (LA-GBS) are common in food-producing animals [7,16,17]. Therefore, the low frequencies of LA-GBS in the database may be related to the relatively high costs of these analyses, which could be unsustainable in the veterinary field. Despite the previous limitations, we considered the dataset of *S. agalactiae* sequences a useful source of information to investigate the molecular epidemiology of resistance genes and identify potential associations and trends, useful to improve surveillance at the human and animal levels.

### 3.2. Epidemiological and Clinical Characteristics

The number of sequences included in the database increased year after year. Indeed, nearly 50% of the overall sequences and more than 50% of the clinical-related sequences were included in the last three years. This increase may be related to the higher feasibility of performing genetic analysis owing to a decrease in cost and availability of new technologies, but an increasing concern with regard to the diffusion of these infections may also be hypothesized.

Europe is the area where about 50% of the sequences originated. When these data are compared to areas with a similar level of health services (Americas and Australia), the frequency observed was much lower, suggesting that the high frequency of European sequences is related to a higher prevalence of GBS infections on the continent. The known high frequency of LA-GBS, particularly in dairy cows [8,18], supports the importance of promoting the registration of LA-GBS sequences in the database to favor the investigation of the measures to reduce the risk of transmission between humans and animals.

Blood represents 25% of overall biological sources of GBS included in the database, while 25% was related to the urinary and intestinal tract (including urine and feces). Milk represents 10% of the whole sequences and only 2.9% of the clinical ones. These data suggest that the role of direct infections through milk in humans is of minor importance compared to the role in dairy cows, where milk is considered the major source of infection [19,20]. Moreover, it can be hypothesized that the pathogenetic characteristics of humans or LA-GBS are different, even if an acquisition of the adaptation gene to an alternative species is common [12,21].

### 3.3. Antimicrobial Resistance Pattern 

The database considers 19 different AMR-genes, but only eight were recovered from the sequences with a proportion >100. Tetracycline resistance in GBS is ubiquitously high (usually >80%), and most GBS strains are characterized by the presence of resistance elements, *tetO* and *tetM* [3]. Indeed, *tetM* is the gene with the higher frequency both in the whole and in the clinical-related database. This gene is related to GBS tetracycline resistance that includes the genes for efflux proteins like TetK and TetL or to ribosomal protection proteins like TetM and TetO. Efflux proteins belong to the major facilitator superfamily (MFS) and all *tet* efflux genes encode membrane-associated proteins that export tetracycline from the cell, reducing the intracellular concentration of the antibiotic. In GBS, these TetK and TetL efflux proteins are encoded by *tetK* and *tetL* genes, respectively, and are usually located on large plasmids or plasmids that can integrate into the bacterial chromosome [22]. These proteins are responsible for detaching tetracycline molecules from the ribosome, thus, the aminoacyl-tRNA molecules can bind again to the ribosomal A-site allowing protein synthesis to continue [23,24]. Since its discovery, tetracycline has been extensively overused, and thus resistance to this antibiotic is now widely observed, as confirmed by the results of this study.

The gene with the second higher frequency is *ermB,* which is related to macrolide resistance. Indeed, the methyl-transferases encoded by the *erm* gene family, composed of more than 40 *erm* variants, represent the most common macrolide resistance mechanism in pathogenic bacteria via ribosomal methylation. *ermB* is considered the most widespread *erm* family gene in Streptococci and GBS [3], as confirmed by this study.

*ant(6)la* has the highest frequency in clinical-related sequences after the previous two genes, and its frequency was significantly higher than expected. This gene is related to resistance to aminoglycosides [25]. The presence of this gene in isolates from human diseases has peculiar importance from a One Health approach because, recently, it was also recovered from streptococci of animal origin [26], supporting the risk of a bi-directional transfer of resistance gene.

The very low frequency of genes related to penicillin-resistance GBS, such as *pbp1a, pbp2a, pbp2b,* and *pbp2x*, confirmed that overall penicillin resistance is still relatively rare [3]. Analogously, a very low frequency of genes related to vancomycin resistance was observed. The resistance is related to the *vanG* gene responsible for altering the vancomycin target site to d-Ala-d-Ser. It was suggested that the lack of vancomycin-sensitivity testing might bias the low frequency of vancomycin resistance isolated due to universal susceptibility to penicillin in GBS strains [3]. However, the analysis of the sequences with very low presence of *van* family genes confirms the low frequency of the vancomycin resistance observed in the field. The same results were observed for gentamycin resistance, associated with *aacA-aphD* gene [27], which was identified in less than four sequences. These data support the evidence of a low frequency for clinical isolates (<0.5%) [1,27]. 

The pathogenetic and the AMR characteristics of pathogens are the results of the combination of their virulence and AMR genes. A cluster analysis was conducted to investigate the pattern of AMR genes in GBS, allowing to identify six different clusters. One cluster (C2) included isolates without AMR genes, while another (C6) included sequences positives for the 10 most frequent AMR genes, even with different proportions. All the other four clusters included sequences with a high proportion of tetracycline gene resistance associated with macrolide (C3), aminoglycosides (C4), and *msrD* gene. This latter one is involved in resistance to azithromycin by acting as a ribosomal protection protein and displacing the macrolides from the ribosome. It is common in other Streptococci, supporting the evidence of AMR gene transmission among different streptococcal species [28].

Sequences included in C5 and C6 have a higher frequency in 2021–2022, and C6 sequences were not found until 2016, whereas C1 and C2 (with lower proportion of AMR genes) had a lower-than-expected frequency in recent years, even not significantly for C2 in clinical isolates. In this latter case, a significant increase in C4 was observed in recent years, suggesting a potential role of this specific resistance mechanism in the development of clinical cases. Moreover, this cluster also has a significantly higher frequency in Asiatic and European isolates, even if the association with clinical cases was confirmed only for asiatic isolates. In this latter continent, a significant increase in C6 was also observed. C2 showed a significantly lower-than-expected frequency in Europe, supporting the previous hypothesis of a role of the acquisition of new resistance genes in the development of GBS disease. It should be noted that Asian sequences provided a contradicting signal. Specifically, both C2 (no AMR) and C6 (high AMR) had a frequency greater than expected. This result suggests that the spread and development of GBS infections in an area with an apparent low prevalence may be due to strains without any significant AMR, but their treatment could lead to an increase in AMR very rapidly and the spread of the resistant strains.

C6 was typically observed in isolates from the uro-vaginal and rectal tract, including urine and feces, as expected. On the other hand, milk was the major source of C2, which supports the very low frequency of clinical isolates from milk, and, therefore, a probable low frequency of antimicrobial treatment.

## 4. Materials and Methods

### 4.1. NCBI Pathogen Detection Isolate Browser and Antibacterial Data

Approximately one million isolates from 53 different bacteria are currently available in the NCBI pathogen detection isolate browser (NPDIB). Therefore, the following parameters were selected to epidemiologically study *S. agalactiae* strains uploaded to this database: scientific name, collection date, location, isolation type, and AMR genotype.

The NPDIB data were downloaded into a Microsoft Excel spreadsheet for this study. The scientific name, collection date, location, isolation type, serovar, and AMR genotype were then organized into columns in a matrix. A sample of *S. agalactiae* was represented by each row of the matrix. 

As retrieved from NPDIB, the AMR genotype data presented the following formatting: “*aac(6′)-Ie/aph(2″)-Ia* = COMPLETE, *catA16* = COMPLETE, *msr(D)* = COMPLETE, *sat4* = COMPLETE, *tet(L)* = COMPLETE”. Before further processing, data were transformed to generate one column for each gene, which was filled with 1 if the gene was discovered in the sample and 0 if it was not. The information in the other columns was changed to align the formats and switch out text entries for numbers.

### 4.2. Statistical Analysis

Data were analyzed on XLSTAT 22.3.1 (Addinsoft, New York, NY, USA, 2022), applying χ^2^ test, Fisher’s exact test, and cluster analysis with the following parameters: Euclidean distance, Ward’s agglomeration method, and truncation with Silhouette index [29].

## 5. Conclusions

The analysis of recorded *S. agalactiae* sequences, even if collected without an epidemiological approach, allows to identify useful information on GBS causing infections in human beings and to identify an important gap represented by the scarce presence of sequences from animal origin, despite the high prevalence of these infections in the animal domain and the known zoonotic and reverse-zoonotic characteristics of these bacteria. Moreover, for the development of effective control programs in a One Health perspective, further study of the pathogenesis involving STs and SNPs would also be of great interest. The recorded data confirm the importance of these infections, as suggested by the large increase in data in the last two years and the increased importance of AMR in the case of GBS infections. Indeed, a cluster of sequence positives for the most frequent AMR genes is prevalent in Asian countries, suggesting the importance of verifying the application of prudent protocols for antimicrobials in areas with an increasing frequency of GBS infections.

## Figures and Tables

**Table 1:** Distribution of *S. agalactiae* sequences in NPDIB by year of reporting.

Year	All		Clinical	
	N	Frequency (%)	N	Frequency (%)
2010–2012	237	13.0	13	1.1
2011–2015	365	20.0	352	30.2
2016–2018	383	21.0	183	15.7
2019–2020	369	20.2	346	29.7
2021–2022	474	25.9	270	23.2
Total	1828	100	1164	100

**Table 2:** Distribution of *S. agalactiae* sequences in NPDIB by geographical area of reporting.

Geographical Area	All		Clinical	
	N	Frequency (%)	N	Frequency (%)
Africa	28	1.5	27	2.3
Americas	147	8.0	136	11.7
Asia w/o Cina	170	9.3	156	13.4
Cina and Hong Kong	169	9.2	147	12.6
Australia	33	1.8	28	2.4
Europe	712	38.9	553	47.5
Non-available	567	31.0	115	9.9
Total	1828	100	1164	100

**Table 3:** Distribution of *S. agalactiae* sequences in NPDIB by biological source.

Biological Source	All		Clinical	
	N	Frequency (%)	N	Frequency (%)
Blood	296	16.2	295	25.3
Brain and cerebrospinal fluid	42	2.3	35	3.0
Human milk	186	10.2	34	2.9
Vagina and rectum	225	12.3	225	19.4
Urine and faeces	81	4.4	79	6.8
Others	137	7.5	128	11.0
Animal	12	0.7	4	0.3
Non-available	849	46.4	364	31.3
Total	1828	100	1164	100

**Table 4:** Distribution of antimicrobial resistance genes in NPDIB *S. agalactiae* sequences.

Gene ^1^	Drug Class	All		Clinical	
		N	Frequency (%)	N	Frequency (%)
*tetL*	Tetracyclines	16	0.9	15	1.3
*tetM*		1094	59.8	774	66.5
*tetO*		250	13.7	147	12.6
*tetS*		14	0.8	13	1.1
*ermA*	Macrolides, lincosamides and streptogramin B	142	7.8	115	9.9
*ermB*		316	17.3	238	20.4
*ermT*		10	0.5	9	0.8
*aac(6′)-Ie-aph(2″)Ia*	Aminoglycosides	41	2.2	39	3.4
*aadE*		134	7.3	80	6.9
*ant(6)la*		162	8.9	149	12.8
*sat4*		46	2.5	45	3.9
*spw*		50	2.7	50	4.3
*mefA*	Macrolides	127	6.9	111	9.5
*msrD*		122	6.7	106	9.1
*catA16*	Phenicol	19	1.0	16	1.4
*catTC*		10	0.5	9	0.8
*lnuB*	Lincosamides	71	3.9	70	6.0
*lsaC*	Lincosamides, streptogramin A, pleuromutilins	22	1.2	18	1.5
*lsaE*		69	3.8	68	5.8

^1^*aph3la, blaTEM, blaTEM116, bleSh, catP, dfrF, lsa, optrA, pbp2x_Q557E, tet44, tetW, vanG, vanR, vanRG, vanTG, vanUG, vanW, vanWG, vanXY, vanYG1*, and *vat* have a frequency in the range 1–4.

**Table 5:** Frequency comparison of the most frequently recovered genes (N > 100) between all the sequences and the clinical ones.

Gene	All	Clinical	Total
*tetM*	46.61%	45.00%	45.93%
*ermB*	13.46%	13.84%	13.62%
*tetO*	**10.65%>** ^1,2^	**8.55%<**	9.76%
*ant(6)la*	**6.90%<**	**8.66%>**	7.65%
*ermA*	6.05%	6.69%	6.32%
*aadE*	5.71%	4.65%	5.26%
*mefA*	5.41%	6.45%	5.85%
*msrD*	5.20%	6.16%	5.61%
Total	100.00%	100.00%	100.00%

^1^ Bold type shows that the observed frequency is different from the expected at Fisher’s exact test (α = 0.05). ^2^ > sign means that an observed frequency higher than expected, while < sign means a lower-than-expected frequency.

**Table 6:** Distribution of the most frequent isolated genes among the six identified clusters.

Cluster	*tetM*	*ermB*	*tetO*	*ant(6)la*	*ermA*	*aadE*	*msrD*	*lnuB*	*lsaE*
**1**	100%	12%	0%	3%	0%	0%	0%	0%	0%
**2**	0%	0%	0%	0%	0%	0%	0%	0%	0%
**3**	86%	0%	6%	4%	97%	0%	0%	0%	0%
**4**	10%	67%	90%	28%	0%	48%	6%	1%	0%
**5**	85%	2%	6%	6%	10%	0%	99%	0%	0%
**6**	62%	72%	33%	91%	10%	29%	30%	100%	100%

**Table 7:** Distribution clusters by year of reporting of all the sequences.

Cluster	2010–2012	2011–2015	2016–2018	2019–2020	2021–2022
1	14.23%	**29.89%>**	**10.20%<**	**18.15%<**	27.52%
2	**19.14%>** ^1,2^	**11.61%<**	**37.42%>**	**11.83%<**	**20.00%<**
3	8.59%	21.88%	23.44%	21.09%	25.00%
4	**5.49%<**	**9.70%<**	**31.65%>**	23.21%	29.96%
5	**4.65%<**	**9.30%<**	**8.14%<**	**41.86%>**	**36.05%>**
6	**0.00%<**	**0.00%<**	15.94%	**62.32%>**	21.74%
Total	12.96%	19.97%	20.95%	20.19%	25.93%

^1^ Bold type means that the observed frequency is different from the expected at Fisher’s exact test (α = 0.05). ^2^ > sign means that an observed frequency is higher than expected, while < sign means a lower-than-expected frequency.

**Table 8:** Distribution clusters by year of reporting of sequences from clinical isolates.

Cluster	2010–2012	2011–2015	2016–2018	2019–2020	2021–2022	Total
**1**	1.07%	**43.69%>** ^1,2^	13.68%	**24.16%<**	**17.41%<**	100.00%
**2**	1.83%	**22.83%<**	19.63%	**22.37%<**	**33.33%>**	100.00%
**3**	0.97%	26.21%	**29.13%>**	26.21%	17.48%	100.00%
**4**	0.72%	**15.22%<**	11.59%	**39.86%>**	**32.61%>**	100.00%
**5**	1.37%	**10.96%<**	9.59%	**49.32%>**	28.77%	100.00%
**6**	0.00%	**0.00%<**	14.71%	**63.24%>**	22.06%	100.00%
**Total**	1.12%	30.24%	15.72%	29.73%	23.20%	100.00%

^1^ Bold type means that the observed frequency is different from the expected at Fisher’s exact test (α = 0.05); ^2^ > sign means that an observed frequency is higher than expected, while < sign means a lo1wer-than-expected frequency.

**Table 9:** Distribution clusters by area of reporting of all the sequences.

Cluster	Area ^1^						
	A	B	C	D	E	F	G
1	2.14%	8.54%	**5.93%<** ^2,3^	**4.15%<**	**2.73%>**	41.40%	35.11%>
2	1.94%	7.78%	**13.82%>**	**11.66%>**	1.30%	**34.34%<**	29.16%
3	0.00%	**18.75%>**	9.38%	**1.56%<**	2.34%	44.53%	23.44%
4	**0.00%<**	**2.11%<**	5.91%	**18.57%>**	0.42%	**51.48%>**	**21.52%<**
5	1.16%	11.63%	11.63%	6.98%	0.00%	**27.91%<**	40.70%
6	0.00%	**0.00%<**	**28.99%>**	**40.58%>**	0.00%	**1.45%<**	28.99%
Total	1.53%	8.05%	9.31%	9.26%	1.81%	38.99%	31.05%

^1^ Area: A—Africa, B—Americas, C—Asia w/o Cina, D—Cina & Honk Kong, E—Australia, F—Europe, G—Not available; ^2^ Bold type means that the observed frequency is different from the expected at Fisher’s exact test (α = 0.05); ^3^ > sign means that an observed frequency is higher than expected, while < sign means a lower-than-expected frequency.

**Table 10:** Distribution clusters by area of reporting of sequences from clinical isolates.

Cluster	Area ^1^						
	A	B	C	D	E	F	G
1	3.20%	11.37%	**8.70%<** ^2,3^	**5.15%**<	3.37%	**60.92%>**	**7.28%<**
2	3.69%	**15.67%>**	**23.96%>**	**20.28%>**	2.30%	**29.49%<**	**4.61%<**
3	0.00%	**22.33%>**	11.65%	**1.94%<**	2.91%	55.34%	5.83%
4	0.00%	**3.62%<**	9.42%	**28.26%>**	0.72%	46.38%	11.59%
5	1.37%	13.70%	13.70%	8.22%	0.00%	**32.88%<**	**30.14%>**
6	0.00%	**0.00%<**	**29.41%>**	**39.71%>**	0.00%	**1.47%<**	**29.41%>**
Total	2.32%	11.70%	13.43%	12.65%	2.41%	47.59%	9.90%

^1^ Area: A—Africa, B—Americas, C—Asia w/o China, D—China & Hong Kong, E—Australia, F—Europe, G—Not available; ^2^ Bold type means that the observed frequency is different from the expected at Fisher’s exact test (α = 0.05); ^3^ > sign means that an observed frequency is higher than expected, while < sign means a lower-than-expected frequency.

**Table 11:** Distribution clusters by the source of reporting of all the sequences.

Cluster	Source ^1^							
	A	B	C	D	E	F	G	H
1	16.25%	2.49%	**1.54%< ^2,3^**	**14.12%>**	4.03%	7.24%	0.36%	**53.97%>**
2	**12.04%<**	1.29%	**23.66%>**	**7.53%<**	3.01%	**9.68%>**	**1.94%>**	**40.86%<**
3	**28.13%>**	3.13%	**0.78%<**	17.19%	3.13%	8.59%	0.00%	39.06%
4	16.46%	3.80%	**26.16%>**	10.55%	3.38%	4.64%	0.00%	**35.02%<**
5	19.77%	0.00%	**0.00%<**	6.98%	6.98%	8.14%	0.00%	**58.14%>**
6	15.94%	2.90%	**0.00%<**	**26.09%>**	**21.74**%**>**	2.90%	0.00%	**30.43%<**
Total	16.19%	2.30%	10.18%	12.31%	4.43%	7.49%	0.66%	46.44%

^1^ Source: A—Blood, B—Brain & cerebrospinal fluid, C—Milk, D—Vagina & rectum, E—Urine & feces, F—Others, G—Animal, H—Not available; ^2^ Bold type means that the observed frequency is different from the expected at Fisher’s exact test (α = 0.05). ^3^ > sign means that an observed frequency is higher than expected, while < sign means a lower-than-expected frequency.

**Table 12:** Distribution clusters by source of reporting of sequences from clinical isolates.

Cluster	Source ^1^							
	A	B	C	D	E	F	G	H
1	24.16%	2.49%	2.31%	21.14%	5.86%	9.77%	0.18%	**34.10%>** ^2,3^
2	25.57%	2.74%	**7.76%>**	15.98%	5.94%	**19.63%>**	**1.37%>**	**21.00%<**
3	**34.95%>**	3.88%	0.97%	21.36%	3.88%	9.71%	0.00%	25.24%
4	28.26%	**6.52%>**	2.17%	18.12%	5.80%	7.97%	0.00%	31.16%
5	23.29%	0.00%	0.00%	**8.22%<**	8.22%	9.59%	0.00%	**50.68%>**
6	16.18%	2.94%	0.00%	26.47%	**22.06%>**	**2.94%<**	0.00%	29.41%
Total	25.34%	3.01%	2.92%	19.33%	6.79%	11.00%	0.34%	31.27%

^1^ Source: A—Blood, B—Brain & cerebrospinal fluid, C—Milk, D—Vagina & rectum, E—Urine & feces, F—Others, G—Animal, H—Not available; ^2^ Bold type means that the observed frequency is different from the expected at Fisher’s exact test (α = 0.05); ^3^ > sign means that an observed frequency higher than expected, while < sign means a lower-than-expected frequency.

## Data Availability

Publicly available datasets were analyzed in this study. This data can be found here: https://www.ncbi.nlm.nih.gov/pathogens/isolates/#taxgroup_name: “Streptococcus%20agalactiae” (accessed on April 2022).

## Supplementary Materials

The following supporting information can be downloaded at: https://www.mdpi.com/article/10.3390/antibiotics11091236/s1; Table S1: Genetic location, molecular functions and drug class resistance induced by antibiotic resistance genes found in *S. agalactiae* genome.

## References

[1] Hays C., Louis M., Plainvert C., Dmytruk N., Touak G., Trieu-Cuot P., Poyart C., Tazi A. (2016). Changing Epidemiology of Group B *Streptococcus* Susceptibility to Fluoroquinolones and Aminoglycosides in France. Antimicrob. Agents Chemother..

[2] Raabe V.N., Shane A.L. (2019). Group B streptococcus (*Streptococcus agalactiae*). Microbiol. Spectr..

[3] Hayes K., O’Halloran F., Cotter L. (2020). A review of antibiotic resistance in Group B *Streptococcus*: The story so far. Crit. Rev. Microbiol..

[4] do Nascimento C.S., Dos Santos N.F.B., Ferreira R.C.C., Taddei C.R. (2019). *Streptococcus agalactiae* in pregnant women in Brazil: Prevalence, serotypes, and antibiotic resistance. Braz. J. Microbiol..

[5] Verani J.R., McGee L., Schrag S.J., Division of Bacterial Diseases N.C.f.I., Respiratory Diseases C.n.f.D.C., Prevention (2010). Prevention of perinatal group B streptococcal disease—Revised guidelines from CDC, 2010. MMWR Recomm. Rep..

[6] Dogan B., Schukken Y.H., Santisteban C., Boor K.J. (2005). Distribution of serotypes and antimicrobial resistance genes among *Streptococcus agalactiae* isolates from bovine and human hosts. J. Clin. Microbiol..

[7] Sora V.M., Panseri S., Nobile M., Di Cesare F., Meroni G., Chiesa L.M., Zecconi A. (2022). Milk Quality and Safety in a One Health Perspective: Results of a Prevalence Study on Dairy Herds in Lombardy (Italy). Life.

[8] Zecconi A., Dell’Orco F., Rizzi N., Vairani D., Cipolla M., Pozzi P., Zanini L. (2019). Cross-sectional study on the prevalence of contagious pathogens in bulk tank milk and their effects on somatic cell counts and milk yield. Ital. J. Anim. Sci..

[9] Soltau J.B., Einax E., Klengel K., Katholm J., Failing K., Wehrend A., Donat K. (2017). Within-herd prevalence thresholds for herd-level detection of mastitis pathogens using multiplex real-time PCR in bulk tank milk samples. J. Dairy Sci..

[10] Delannoy C.M.J., Samai H., Labrie L. (2021). *Streptococcus agalactiae* serotype IV in farmed tilapia. Aquaculture.

[11] Tavares G.C., de Queiroz G.A., Assis G.B.N., Leibowitz M.P., Teixeira J.P., Figueiredo H.C.P., Leal C.A.G. (2018). Disease outbreaks in farmed Amazon catfish (*Leiarius marmoratus* x *Pseudoplatystoma corruscans*) caused by *Streptococcus agalactiae*, *S. iniae*, and *S. dysgalactiae*. Aquaculture.

[12] Crestani C., Forde T.L., Lycett S.J., Holmes M.A., Fasth C., Persson-Waller K., Zadoks R.N. (2021). The fall and rise of group B *Streptococcus* in dairy cattle: Reintroduction due to human- to- cattle host jumps?. Microb. Genom..

[13] Sorensen U.B.S., Klaas I.C., Boes J., Farre M. (2019). The distribution of clones of *Streptococcus agalactiae* (group B streptococci) among herdspersons and dairy cows demonstrates lack of host specificity for some lineages. Vet. Microbiol..

[14] Castor M.L., Whitney C.G., Como-Sabetti K., Facklam R.R., Ferrieri P., Bartkus J.M., Juni B.A., Cieslak P.R., Farley M.M., Dumas N.B. (2008). Antibiotic resistance patterns in invasive group B streptococcal isolates. Infect. Dis. Obstet. Gynecol..

[15] Ferri M., Ranucci E., Romagnoli P., Giaccone V. (2017). Antimicrobial resistance: A global emerging threat to public health systems. Crit Rev Food Sci Nutr.

[16] Pang M.D., Sun L.C., He T., Bao H.D., Zhang L.L., Zhou Y., Zhang H., Wei R.C., Liu Y.J., Wang R. (2017). Molecular and virulence characterization of highly prevalent *Streptococcus agalactiae* circulated in bovine dairy herds. Vet. Res..

[17] Lyhs U., Kulkas L., Katholm J., Waller K.P., Saha K., Tomusk R., Zadoks R. (2016). *Streptococcus agalactiae* Serotype IV in Humans and Cattle, Northern Europe. Emerg. Infect. Dis. J..

[18] Mweu M.M., Nielsen S.S., Halasa T., Toft N. (2012). Annual incidence, prevalence and transmission characteristics of *Streptococcus agalactiae* in Danish dairy herds. Prev. Vet. Med..

[19] Zecconi A. (2007). Contagious mastitis control. FIL-IDF Bull..

[20] Deng Z.J., Koop G., Hogeveen H., Fischer E.A.J., van den Borne B.H.P., van der Tol R., Lam T. (2021). Transmission dynamics of *Staphylococcus aureus* and *Streptococcus agalactiae* in a Dutch dairy herd using an automatic milking system. Prev. Vet. Med..

[21] Botelho A.C.N., Ferreira A.F.M., Fracalanzza S.E.L., Teixeira L.M., Pinto T.C.A. (2018). A Perspective on the Potential Zoonotic Role of *Streptococcus agalactiae*: Searching for a Missing Link in Alternative Transmission Routes. Front. Microbiol..

[22] Chopra I., Roberts M. (2001). Tetracycline antibiotics: Mode of action, applications, molecular biology, and epidemiology of bacterial resistance. Microbiol. Mol. Biol. Rev..

[23] Trieber C.A., Burkhardt N., Nierhaus K.H., Taylor D.E. (1998). Ribosomal protection from tetracycline mediated by Tet(O): Tet(O) interaction with ribosomes is GTP-dependent. Biol. Chem..

[24] Connell S.R., Tracz D.M., Nierhaus K.H., Taylor D.E. (2003). Ribosomal protection proteins and their mechanism of tetracycline resistance. Antimicrob. Agents Chemother..

[25] Berbel D., Càmara J., García E., Tubau F., Guérin F., Giard J.C., Domínguez M., Cattoir V., Ardanuy C. (2019). A novel genomic island harbouring *lsa(E)* and *lnu(B)* genes and a defective prophage in a *Streptococcus pyogenes* isolate resistant to lincosamide, streptogramin A and pleuromutilin antibiotics. Int. J. Antimicrob. Agents.

[26] Wang S.J., Zhang D.F., Jiang C.G., He H.J., Cui C.C., Duan W.T., Hu S.P., Wang J., Cai X.H. (2021). Strain Characterization of *Streptococcus suis* Serotypes 28 and 31, Which Harbor the Resistance Genes optrA and ant(6)-Ia. Pathogens.

[27] Doumith M., Mushtaq S., Martin V., Chaudhry A., Adkin R., Coelho J., Chalker V., MacGowan A., Woodford N., Livermore D.M. (2017). Genomic sequences of *Streptococcus agalactiae* with high-level gentamicin resistance, collected in the BSAC bacteraemia surveillance. J. Antimicrob. Chemother..

[28] Varaldo P.E., Montanari M.P., Giovanetti E. (2009). Genetic Elements Responsible for Erythromycin Resistance in Streptococci. Antimicrob. Agents Chemother..

[29] Everitt B.S., Landau S., Leese M., Stahl D. (2011). Cluster Analysis.

