# Supplementary material for: Epidemiology of Antimicrobial Resistance Genes in Streptococcus agalactiae Sequences from a Public Database in a One Health Perspective
Source: Antibiotics (Basel). 2022 Sep 12;11(9):1236. doi: 10.3390/antibiotics11091236 (PMC9495180; doi:10.3390/antibiotics11091236)
Supplement: Supplementary file 1 [file antibiotics-11-01236-s001.zip › Supplementary Table S1.pdf]

Table S1. Genetic location, molecular functions and drug class resistance induced by antibiotic resistance genes found in *Str.agalactiae* genome.

Gene	Location in genome	Molecular function	Drug class
tetM	Mobile element (transposon Tn916 family)	Antibiotic target protection	Tetracycline
tetW	Mobile element (conjugative and non conjugative plasmid)		
tetO[1]	Chromosome		
tetS	Chromosome		
tet(44)	Mobile element		
tetK	Mobile element (conjugative plasmid)	Antibiotic efflux	
tetL	Chromosome		
ermA, ermB[1]*, ermT*	Mobile element (conjugative plasmid pIP501 or ICESag37, or transposon Tn917)	Ribosomal alteration	Macrolides, lincosamides and streptogramin B (MLS_B)[2]
ant(6)la, aadE	Chromosome/plasmid	Antibiotic inactivation	Aminoglycosides
aac(6')-ie-aph(2'')-Ia	Plasmid/transposon		
Aph(3')-Ia	Chromosome		
sat-4	Mobile element (plasmid)		
spw	Mobile element (plasmids and transposons)		
mefA[3]*, msrD*	Mobile element (Conjugative transposons Tn1207.1 or Tn1207.3)	Antibiotic efflux	Macrolides
catA16, catTC	Mobile element (conjugative plasmid pIP501 or transposon)	Antibiotic inactivation	Phenicol
catP	Chromosome and transposon		
lnuB	Chromosome	Antibiotic inactivation	Lincosamides (L phenotype)
Isa, IsaC, IsaE	Mobile element (transposon)	Antibiotic efflux	Lincosamides, streptogramin A, pleuromutilins (LSA and LSAP phenotypes)
blaTEM, blaTEM116	Mobile element (plasmid)	Antibiotic inactivation	β-lactam
PBP2x, pbp2x_Q557E	Chromosome	Antibiotic target alteration	
bleSh	Transposon	Antibiotic target alteration	Glycopeptide
vanG, vanRG[4]*, vanTG*[4]*, vanUG*[4]*, vanWG*[4]*, vanYG1*[4]	Chromosome		
vanR[5]	Chromosome		
vanXY	Chromosome		
vanW[6]	unknown	unknown	

dfrF	Chromosome	Antibiotic target replacement	Diaminopyrimidine
optrA	Plasmid	antibiotic target protection	Oxazolidinone, phenicol
vat	Mobile element	Antibiotic inactivation	Streptogramin

[1] Tetracycline resistance genes are often found on the same mobile element as erythromycin resistance genes. [2] This resistance can be constitutive (cMLSB) resistance, or (iMLSB) resistance; iMLSB is mediated by mef gene. [3] Confers resistance to 15-membered ring macrolides (M phenotype) only, while erm(B) determines cross-resistance to all macrolides, lincosamides, and streptogramins B. [4] are all variant found in the *vanG* gene cluster. [5] Is a OmpR-family transcriptional activator in the VanSR regulatory system, after activation by *VanS*, it promotes cotranscription of *VanA*, *VanH*, and *VanX*. [6] Found in vancomycin resistance gene clusters *vanB* and *vanG*.
